# Biochemical, Histological, and Transcriptomic Analyses Reveal Underlying Differences in Flesh Quality between Wild and Farmed Ricefield Eel (*Monopterus albus*)

**DOI:** 10.3390/foods13111751

**Published:** 2024-06-03

**Authors:** Hang Yang, Quan Yuan, Mohammad Mizanur Rahman, Weiwei Lv, Weiwei Huang, Wei Hu, Wenzong Zhou

**Affiliations:** 1 Eco-Environmental Protection Research Institute, Shanghai Academy of Agricultural Sciences, Shanghai 201403, China; yanghangqu@foxmail.com (H.Y.); quanyuan2016@126.com (Q.Y.); wwlv1986@sina.com (W.L.); hwwswx@163.com (W.H.); 2 Key Laboratory of Integrated Rice-Fish Farming Ecosystem, Ministry of Agriculture and Rural Affairs, Shanghai Academy of Agricultural Sciences, Shanghai 201403, China; 3 Institute of Marine Sciences, University of Chittagong, Chattogram 4331, Bangladesh; mizan@cu.ac.bd; 4 State Key Laboratory of Freshwater Ecology and Biotechnology, Institute of Hydrobiology, Chinese Academy of Sciences, Hubei Hongshan Laboratory, Wuhan 430072, China; huwei@ihb.ac.cn

**Keywords:** *Monopterus albus*, flesh quality, transcriptome, integrated analysis

## Abstract

The present study aimed to systematically investigate the underlying differences in flesh quality between wild and farmed *Monopterus albus*. Fifteen healthy *M. albus* per group with an average body weight of 45 g were sampled to analyze muscle parameters by biochemical indicators, histomorphology, and molecular biology. Compared with the wild fish, the farmed *M. albus* in flesh had lower crude protein, collagen, lysine, histidine, total amino acids, SFA, n-3 PUFA contents, and n-3/n-6 ratio (*p* < 0.05), and higher moisture, crude lipid, crude ash, MUFA, n-6PUFA, and total PUFA contents (*p* < 0.05). The thawing loss, drip loss, steaming loss, and boiling loss in the farmed group were significantly higher, and hardness, springiness, cohesiveness, gumminess, chewiness, and resilience were significantly lower than those in the wild group (*p* < 0.05). In addition, higher muscle fiber density and lower muscle fiber diameter were observed in wild *M. albus* (*p* < 0.05). In muscle transcriptome profiling, differentially expressed genes and enriched pathways are primarily associated with muscle development, protein synthesis, catabolism, lipid metabolism, and immunity. To the best of our knowledge, this is the first investigation that compares the flesh quality between wild and farmed *M. albus* in terms of biochemistry, histology, and molecular biology levels. Overall, wild *M. albus* had a higher nutritional value and texture quality than farmed *M. albus*.

## 1. Introduction

The global demand for fish has been accelerating owing to the increased population and awareness of the health benefits of fish consumption instead of meat products. The main sources of fish are natural resources (i.e., artificial fishing) and aquaculture [1]. However, the increase in total fish consumption and unsustainable fishing operations has made it impossible to meet the growing demand with wild-caught fish alone [2], and the aquaculture industry has become a suitable means to satisfy the global fish supply [3]. In recent years, the wide acceptance of artificial compound feeds coupled with the continuous optimization of nutritional balancing has led to an increase in farmed fish production year by year [4]. However, with the increase in production, the problem of the poor quality of farmed fish has seriously affected the healthy development of the aquaculture industry, including market prices and export restrictions. Since there are differences in the flesh quality between wild and cultured fish, consumers are more inclined to accept the former, even if it comes with a higher price tag [5].

Fish flesh quality is a complex and comprehensive assessment system. While considering the important role of the muscle in the organism, fish flesh quality assessment mainly involves the various nutrient contents, tissue morphology, texture quality, water holding capacity, and so on, which covers a wide range of evaluation indicators [6]. Factors that cause differences in flesh quality between wild and aquaculture fish include the living environment (water temperature, water quality, and water salinity), developmental stage, dietary sources, and exercise intensity [7]. Compared to cultured fish, wild fish live in environments with high dissolved oxygen, low living density, and low ammonia–nitrogen levels, which is less stressful for fish, thereby favoring muscle quality; however, excess heavy metal contents in the wild environment may also accumulate in fish muscle [8]. In addition, wild fish have a richer diet composition under natural conditions; the food they consume is easily digested, which influences flesh quality, and moderate exercise can also lead to improved flesh texture [9].

The Asian swamp eel *Monopterus albus*, known as rice field eel, is an important traditional aquaculture species in China, with a culture production of 334 thousand tons in 2022 [10]. This species is preferred due to its tasty flesh and high nutritional and medicinal value. However, recently, a few studies have reported on the flesh quality of wild and cultured fish of Atlantic salmon *Salmo salar* [11], sea bass *Dicentrarchus labrax* [9], Nile tilapia *Oreochromis niloticus* [12], Coilia nasus *Engraulidae* [13], blackspot seabream *Pagellus bogaraveo* [14], large yellow croaker *Larimichthys crocea* [15], and black rockfish *Sebastes schlegelii* [16]; the studies mainly focused on muscle nutritional composition and textural properties, while information on molecular biology was poorly investigated. Muscle quality is a comprehensive characteristic system which is difficult to evaluate in terms of only a few biochemical parameters.

Transcriptome profiling is a nascent but powerful technology that continuously helps in our understanding of fish life forms. This technology plays a vital role in understanding the molecular mechanisms of growth, flesh quality, and immunity in response to altered environment and nutrition and helps to identify and quantify the differentially expressed genes in fish [17,18]. Recently, an increasing number of transcriptome analyses have been performed in a variety of commercially important aquatic organisms [19,20,21,22,23]. However, few transcriptome studies have been conducted in *M. albus*, and no information is available on the transcriptome of *M. albus* in response to different life modes. Therefore, this study aimed to analyze the relationship among the basic nutritional indicators, texture profile, water holding capacity, and transcriptome of *M. albus* from biochemistry, histology, and molecular biology levels in order to systematically compare the flesh quality of wild and aquaculture *M. albus*. The results provide a theoretical basis for the subsequent upgrading of healthy aquaculture technology and nutrient regulation of the flesh quality of cultured *M. albus*.

## 2. Materials and Methods

The Animal Ethics Committee of the Shanghai Academy of Agricultural Sciences approved all animal procedures.

### 2.1. Sample Collection

In this work, healthy *M. albus* of wild (*n* = 15) and farmed (*n* = 15) sources were collected, with an average body weight of 45.3 ± 5.1 g and 44.2 ± 3.8 g, from Jinshan district, Shanghai (N 30.78, E 121.18), and the Zhuanghang Comprehensive Experiment Station of the Shanghai Academy of Agricultural Sciences (N 30.89, E 121.41), respectively. The farmed *M. albus* with an initial body weight of 15.2 ± 0.5 g were fed a commercial diet containing 43% crude protein and 7% crude lipid at 16:00 each day by hand for 10 weeks. During the feeding trial, the water was continuously aerated, and one-fourth of the aeration water was replaced every five days (dissolved O^2^ ≥ 5.8 mg/L, water temperature 28 ± 2 °C, pH 7.3 ± 0.2, NH^4+^–N < 0.5 mg/L).

The body surface of *M. albus* was wiped with 75% ethanol to collect muscle samples, and both sides of the muscle were sampled with a sterilized scalpel. A portion was removed to determine the water holding capacity and texture profile analysis; another portion was placed in liquid nitrogen for transcriptome analysis and qRT-PCR verification; and the other portion was collected and fixed in 4% formaldehyde for observing muscle morphology. Finally, the rest of the flesh was taken and stored frozen at −20 °C for muscle proximate composition, fatty acid, and amino acid profiles.

### 2.2. Sample Analysis

#### 2.2.1. Muscle Nutrient Composition

The muscle proximate composition was analyzed according to AOAC methods [24]. Moisture was measured by the drying method at 105 ± 5 °C to constant weight. The crude ash was determined using the burning method at 550 °C for 6 h. The crude lipid was analyzed by the chloroform–methanol method. The crude protein was carried out by the Kjeldahl method, and the content was calculated by N × 6.25. The total hydroxyproline content was determined by the alkaline hydrolysis method using a kit produced by Nanjing Jiancheng Bioengineering Institute (Nanjing, China) according to the instructions, which was multiplied by a conversion factor of 8 to obtain the total collagen content [25].

In terms of the amino acid profile, freeze-dried muscle samples were weighed to 40–50 mg and hydrolyzed in 6 mol/L hydrochloric acid solution at 110 °C for 24 h. After hydrolysis, 1 mL of the hydrolysate was taken into the injection vial and analyzed by the Hitachi L-8900 automatic amino acid analyzer (Tokyo, Japan) [26].

The fatty acid profile was determined using a GC-MS gas chromatograph (Agilent 7980B, City of Santa Clara, CA, USA). Muscle samples of 0.3–0.5 g were placed in a 10 mL tube, and 5 mL of methanol/chloroform (1:2) was added to homogenize with a high-speed disperser. The mixture was filtered, and 4 mL of distilled water was added to the centrifuge (Cence HT160, Changsha, China) for 5 min at 4 °C (2300 g), and then the supernatant was removed to concentrate the lower layer. Subsequently, 1 mL of chromatographically pure n-hexane was added to dissolve the lipid, and 1 mL of 0.4 mol/L potassium hydroxide–methanol solution was added for 30 min to carry out methyl esterification, followed by the addition of 2 mL of deionized water, and then the upper layer of the solution was extracted after being separated and detected and analyzed by gas chromatography, and the contents of the various fatty acids were calculated according to the area-normalized method [27].

#### 2.2.2. Muscle Quality Analysis

The flesh texture profiles analysis (TPA) was measured using the TA.XTPlus texture analyzer (Stable Micro Systems, Godalming, UK). Dorsal muscles above the lateral line were cut into cubes of 1.0 cm × 1.0 cm × 0.5 cm. The test conditions were as follows: a 25 mm × 25 mm flat-bottomed cylindrical probe, a descent speed of 2 mm/s, a test speed of 1 mm/s, a test interval of 5 s, and a compression ratio of 30% [26]. The TPA tests were performed at room temperature.

#### 2.2.3. Muscle Tissue Morphology

The muscle samples were fixed in 4% formaldehyde for at least 48 h and then dehydrated with ethanol, transparent with xylene, embedded in paraffin, cut into 5 μm longitudinal sections (Leika RM 2235, Wetzlar, Germany), and stained with hematoxylin–eosin (HE). The sections were photographed under an optical microscope to observe the muscle morphology parameters (Nikon YS100, Tokyo, Japan). The muscle fiber diameter and muscle fiber density were measured and calculated according to Yang et al. [28]. The water holding capacity (thawing loss, drip loss, steaming loss, and boiling loss) was measured by the same method used in the study of Xu et al. [29].

#### 2.2.4. Transcriptome Analysis

Transcriptome sequencing (RNA-Seq) was conducted by Shanghai Majorbio Bio-pharm Technology Co., Ltd. (Shanghai, China). The muscles of five fish per group were pooled as 1 sample, with a total of 15 samples to perform the transcriptomic assay. A transcriptome sequencing cDNA library was established by the TruSeq RNA Sample Preparation Kit (Illumina, San Diego, CA, USA), and high-throughput sequencing was carried out using the Illumina HiSeq 4000 sequencing platform. Fast QC software (version 0.11.9) was used to assess the quality of the raw sequencing data, and the low-quality fragments in the raw data were removed to ensure that the data obtained were of a high quality and valid sequencing data (clean reads). The annotated genes were evaluated for their gene expression level by the FPKM method, with the farmed group as the control and the wild group as the experimental group. Differentially expressed genes (DEGs) were identified using DEGSeq software (Version 1.38.0), and the threshold was FDR < 0.05 and |log2(FC)| > 1. Based on the results of DEGs, GO (gene ontology) functional annotation and KEGGs (Kyoto Encyclopedia of Genes and Genomes) signal pathway enrichment analyses of DEGs were carried out using a hypergeometric distribution method.

#### 2.2.5. DEGs Verification Using qRT-PCR

Six genes were used to analyze qRT-PCR in order to validate the RNA-Seq results. The PCR primers were obtained and designed based on *M. albus* sequences in the GenBank accession (Table S1). Total RNA for muscle samples was extracted by using Trizol reagent (Thermo Fisher Scientific, Waltham, MA, USA), and the concentration and purity of RNA were detected by a UV spectrophotometer and agarose gel electrophoresis. Then, the RNA was reverse-transcribed into cDNA using the PrimeScript^TM^ RT reagent Kit (Takara, Dalian, China). The reaction program of real-time quantitative PCR was as follows: pre-denaturation at 95 °C for 30 s, 35 cycles of denaturation at 95 °C for 5 s, annealing at 58 °C for 15 s, and extension at 72 °C for 20 s; finally, the melting curve was used to confirm the specificity. The relative expression levels in muscle tissues were calculated by the 2^−ΔΔCt^ method [28].

### 2.3. Statistical Analysis

The experimental results of the biochemical parameters were expressed as the mean with standard deviation. All the data of the biochemical parameters were subjected to equality of variances with Levene’s test with SPSS software (Version 22.0), and independent sample t-tests were performed to evaluate significant differences (at least for *p* < 0.05) between wild and farmed *M. albus*.

## 3. Results

### 3.1. Muscle Proximate Composition

In Table 1, it is shown that the wild *M. albus* muscle had higher contents of crude protein and collagen (*p* < 0.05) but lower contents of moisture, crude lipid, and crude ash than farmed *M. albus* (*p* < 0.05).

### 3.2. Muscle Amino Acid Composition

Compared with farmed fish, wild *M. albus* showed lower levels of lysine, histidine, aspartate, glycine, alanine, tyrosine, cysteine, glutamate, proline, and total amino acids, and a higher serine content in the flesh (Table 2).

### 3.3. Muscle Fatty Acid Composition

A total of 16 and 13 fatty acids were detected in the muscle of wild and farmed fish, respectively, and there were significant differences in all the fatty acids between the wild and farmed groups, except for C16:1. Table 3 shows higher contents of C11:0, C12:0, C13:0, C15:0, C16:0, C18:0, C23:0, C24:1, C20:4, C20:5, C22:6, SFAs, n-3 PUFAs, and n-3/n-6 and lower levels of C14:0, C16:1, C17:1, C18:1, C18:2, C18:3, MUFAs, n-6 PUFAs, and total PUFAs in the wild *M. albus* group than those in the farmed group (*p* < 0.05).

### 3.4. Muscle Water Holding Capacity, Texture Profile, and Muscle Histology

As shown in Table 4, the muscle thawing loss, drip loss, steaming loss, and boiling loss in wild *M. albus* were found to be significantly lower than in farmed *M. albus* (*p* < 0.05).

Hardness, springiness, cohesiveness, gumminess, chewiness, and resilience in the flesh of wild fish showed significantly higher values than those of farmed fish (*p* < 0.05).

**Figure 1:**
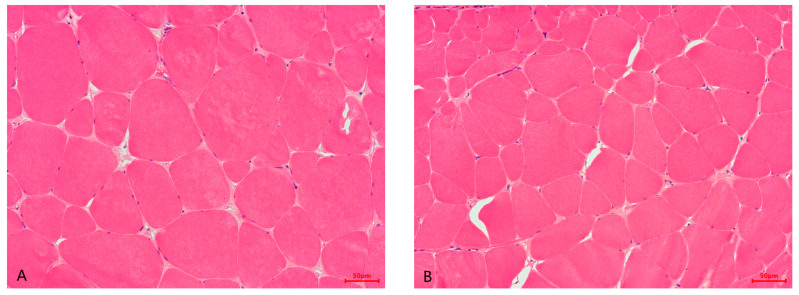

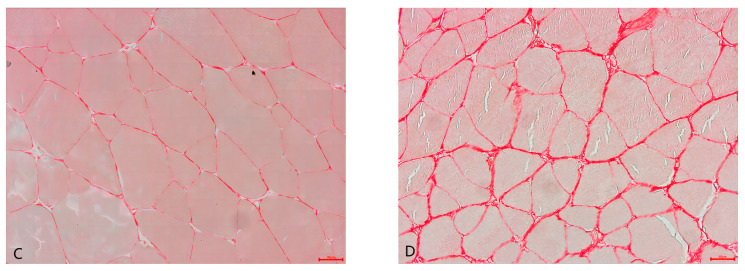
The muscle histological structure of the wild and farmed *M. albus* (200×). (**A**) farmed *M. albus* (HE staining); (**B**) wild *M. albus* (HE staining); (**C**) farmed *M. albus* (sirius red staining); (**D**) wild *M. albus* (sirius red staining).

Muscle morphology for HE staining and sirius red staining of wild and farmed groups is illustrated in Figure 1.

Lower muscle fiber diameter and higher muscle fiber density were observed in wild *M. albus* (*p* < 0.05). (Table 4; Figure 1A,B).

In Figure 1C,D, the red lines among the muscle fibers represent collagen, and as can be seen, the distribution area of collagen in the intermuscular of the wild group was larger than the farmed group.

### 3.5. Transcriptomic Assay

**Figure 2:**
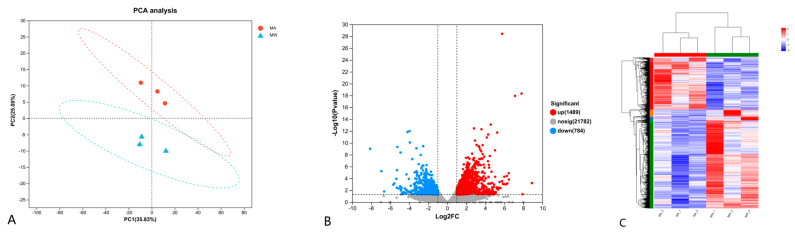
The transcriptomic features in muscles of the wild and farmed *M. albus*. (**A**) The PCA analysis for the transcriptomics profiles; (**B**) volcano plot of muscle differentially expressed genes; (**C**) heatmap visualization of differentially expressed genes.

As shown in Figure 2A, there was no significant overlap between the wild and farmed groups in the PCA analysis. Compared with the farmed group, a total of 2273 differentially expressed genes (DEGs) were identified in the wild group, including 1489 upregulated and 784 downregulated DEGs, or 65.51% and 34.49% of the total number of genes, respectively (Figure 2B). The DEGs in the two groups were clustered and analyzed, as can be seen in Figure 2C; the six samples were clustered into two categories (MA1, MA2, and MA3; MW1, MW2, and MW3), indicating that these aggregated genes may have similar functional annotations or be in the same metabolic pathway, and there are obvious expression differences between the samples from different groups.

**Figure 3:**
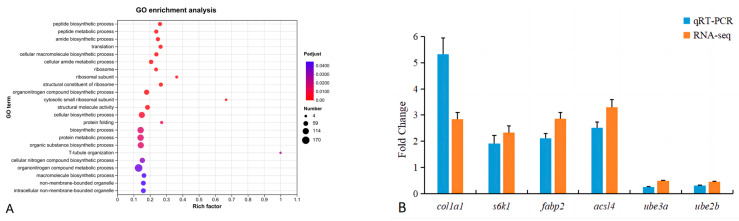
Scatterplot of enriched GO terms for differentially expressed genes (**A**) and results of qRT-PCR verification (**B**). *col1a1*, collagen alpha-1(I); *s6k1*, ribosomal protein S6 kinase beta-1; *fabp2* fatty acid binding protein 2; *acsl4*, acyl-CoA synthetase long-chain family member 4; *ube3a*, ubiquitin–protein ligase E3A; *ube2b*, ubiquitin conjugating enzyme E2 B.

To further investigate the functions of DEGs in the farmed and wild groups, GO functional enrichment analysis was performed for DEGs, which were enriched in the cellular component (CC), molecular function (MF), and biological process (BP). Ribosome, ribosomal subunit, cytosolic small ribosomal subunit, non-membrane-bounded organelle, and intracellular non-membrane-bounded organelle were significantly enriched in the CC; two terms of the structural constituent of ribosome and structural molecule activity were enriched in MF. Sixteen terms in BP were significantly enriched in peptide biosynthesis processes, peptide metabolism processes, amide biosynthesis processes, and so on (Figure 3A).

As shown in Table 5, all the DEGs were subjected to KEGGs enrichment analysis, revealing that a total of 39 metabolic pathways were significantly enriched (*p* < 0.05). The pathways were mainly related to muscle development, extracellular matrix, lipid metabolism, amino acid metabolism, and immune metabolism.

### 3.6. qRT-PCR Verification

As shown in Figure 3B, the mRNA of *col1a1*, *s6k1, fabp2*, and *acsl4* was upregulated, and that of *ube3a* and *ube2b* was downregulated in the qRT-PCR results, which is consistent with the RNA-seq results, indicating that the RNA-seq results were reliable.

## 4. Discussion

Moisture, crude lipid, and crude protein are the basic indicators used for evaluating the nutritional level and quality of fish fillet. The proximate composition is influenced by a number of factors such as fish size, species, temperature, production system, and feeding [30]. In this study, the moisture and crude lipid of muscle were significantly lower, while crude protein and collagen were significantly higher in the wild group compared to the farmed group. This result is the same as that obtained on sea bass [31], Atlantic salmon [11], and blackspot seabream [14]. The higher crude lipid level in farmed *M. albus* may be due to the high lipid and starch contents of commercial feed and farmed space constraints coupled with relatively reduced exercise, resulting in increased fat accumulation. The food caught by wild fish is palatable and has a high conversion utilization rate, leading to a high rate of muscle crude protein deposition. At the same time, proper exercise can also increase the flesh collagen content [32]. Studies have shown that moisture content affects muscle quality in fish; the lower the moisture content, the higher the nutritional level [33]. Therefore, in terms of basic nutritional indicators, wild *M. albus* are superior to farmed ones.

The protein quality mainly depends on the type of composition and the amino acids content [34]. In this experiment, the muscle histidine and lysine contents in the essential amino acids of wild *M. albus* were higher than those of farmed *M. albus*. Relative to the essential amino acids, wild fish had significantly higher levels of a variety of non-essential amino acids, such as aspartate, glycine, alanine, tyrosine, cysteine, glutamate, and proline, as well as a significantly higher total amino acids content. The reasons for those differences in amino acids between wild and farmed *M. albus* may be as follows: one reason is the difference in food composition, as the diet of farmed fish is higher in plant protein sources, but wild fish feed is mainly derived from natural animal bait. The amino acid composition and digestibility of the different protein sources vary greatly, affecting the deposition of amino acids in the muscle of fish [35]. Among them, the histidine and lysine levels are higher in animal bait, which may be the reason for the higher contents of these two essential amino acids in the muscles of the wild group. Another reason for this is that they may be affected by the growing environment: wild fish may be in a state of starvation or non-satiation for a long period of time, and the catabolism of non-essential amino acids can play a role in energy supply [36], leading to the fact that most of the non-essential amino acids in the muscles of the wild group are higher than those of the farmed group.

Fish muscle is considered to have beneficial effects on human health due to its richness in polyunsaturated fatty acids [37]. In the present experiment, the farmed fish had higher saturated fatty acids (SFAs) and lower monounsaturated (MUFAs) and polyunsaturated fatty acids (PUFAs), again closely related to the living environment and dietary composition. The unsaturated fatty acids in the commercial feed were enhanced by the addition of soya bean oil and fish oil, together with aquatic animal as well as plant-based ingredients, resulting in a higher proportion of unsaturated fatty acids deposited in the muscle of *M. albus* raised on commercial feed and a lower proportion of saturated fatty acid content. In addition, the high contents of eicosapentaenoic acid (EPA) and docosahexaenoic acid (DHA) in the flesh of the wild group may be attributed to the fact that the increase in long-chain unsaturated fatty acids in the fish improves the membrane lipid fluidity, and that the fish grown in the wild environment lead to an increase in the content of n-3PUFAs in the body compared to the farmed fish in order to better cope with the variable environment [38]. However, the evaluation of muscle nutritional value through fatty acids cannot be assessed using simply total fatty acids content. PUFAs include n-6 PUFAs and n-3 PUFAs, and studies have shown that excessive n-6 PUFA causes lipid metabolism disorders, inflammation, and oxidative stress damage, which are risk factors for cardiovascular disease [39]. The n-3 PUFAs have important physiological functions in the human body, especially EPA and DHA, which play the role of regulating inflammatory and immune processes as well as preventing cardiovascular disease, while is also an important indicator of the nutritional quality of fish [40]. In terms of the flesh quality evaluation index, the ratio of n-3/n-6 or n-6/n-3 PUFAs is more appropriate than single n-3, n-6, or total PUFAs. In the present study, the muscle n-3/n-6 ratio of wild fish was significantly higher than that of farmed fish, suggesting that the former had higher lipid nutrition than the latter, and similar reports were found in trials comparing wild and farmed fish, including pintado *Pseudoplatystoma corruscans* and pacu *Piaractus mesopotamicus* fish [41].

Flesh texture is an important attribute reflecting consumer satisfaction and the mechanical processing of fish fillets, and a more objective method of evaluating fish texture is usually through texture instrumentation [42]. The instrumental measurement and analysis of texture can reduce errors caused by the evaluation of human factors, thus showing more accurate results, which are often described in terms of TPA (texture profile analysis) patterns (including hardness, springiness, cohesiveness, chewiness, etc.) to describe textural attributes. Usually, softer fillets can reduce consumer acceptability, which is one of the main reasons why consumers prefer wild fish [43]. Textural parameters such as muscle hardness, springiness, and chewiness were significantly higher in wild *M. albus* than in farmed ones, and similar results were seen in Atlantic salmon [11], blackspot seabream [14], and small yellow croaker *Larimichthys polyactis* [44].

Muscle textural properties are mainly influenced by muscle collagen content, myofiber density, muscle water holding capacity, and lipid content [45]. In fish, muscle fibers are arranged in bundles along the anterior–posterior axis, constituting muscle segments, which are surrounded by an extracellular matrix called the epimysium, perimysium (PM), and endomysium (EM), respectively. In Figure 2, the PM and EM can be clearly observed. The extracellular matrix consists of collagen, elastin, proteoglycan, etc., of which collagen is the most important component [46]. Collagen is mainly responsible for driving the muscle fibers, while it also determines the morphology of the muscle and protects it from overstretching. In fish or other aquatic animals, muscle collagen content is positively correlated with flesh texture [47]. Therefore, collagen, which constitutes the intermuscular connective tissue, is one of the main indicators of fish flesh quality. Myofibrils are the basic units of the muscle, and the number and size distribution of myofibrils are mainly influenced by fish species, growth stage, feed, exercise, and environmental factors, which are important factors in determining the textural characteristics of fish [45]. It has been shown that the density of muscle fibers is positively correlated with muscle hardness, springiness, cohesiveness, and chewiness [48]. Moisture is the most dominant component of fish, typically making up to 70 to 80% of the muscle, which can affect the quality and processing of fish [49]. Water holding capacity is usually used to measure the ability of muscle tissue to retain water, which not only affects muscle color, aroma, flavor, nutrient content, juiciness, hardness, and other edible qualities but also directly influence the economic value of flesh. Studies in fish have shown that the moisture content of muscle is negatively correlated with shear force [50]. Lipid has an important effect on the nutritional value and eating qualities of fish fillet, including texture and flavor. Regarding the relationship between muscle lipid content and muscle texture, a study displayed that muscle lipid was positively correlated with juiciness and negatively correlated with hardness [51]. In this study, the collagen content (Table 1; Figure 1), muscle fiber density (Table 4; Figure 1), and water holding capacity, including thawing loss, drip loss, steaming loss, and boiling loss (Table 4), were higher in the wild group than in the farmed group, indicating that the flesh texture of the former was superior to that of the latter. Similar results with the present study were found in wild and farmed fish, such as blackspot seabream [14], common sole *Solea solea* [52], and small yellow croaker [44].

To further investigate the mechanism of the differences in flesh quality between wild and farmed *M. albus*, transcriptomic analyses were performed in this study. A total of 39 significantly enriched pathways were screened in the transcriptome results. Among them, the pathways related to muscle quality and muscle development included Ribosome, Protein processing in endoplasmic reticulum, the PPAR signaling pathway, glycerolipid metabolism, peroxisome, mTOR signaling pathways, ECM–receptor interaction, and ubiquitin-mediated proteolysis.

The ribosome is the main site of protein synthesis in the cell, and the process of RNA translation to protein is completed in the ribosome. Ribosomal proteins control gene transcription and mRNA translation, affect cell differentiation, proliferation, and apoptosis, and play an important role in muscle growth, development, and protein synthesis [53]. In this study, the signaling pathways regarding protein synthesis such as ribosome and protein processing in endoplasmic reticulum were significantly enriched, in which 40 out of 42 differential genes in the ribosomal pathway were significantly upregulated (Table S2).

Skeletal muscle protein deposition or growth is a dynamic process of continuous protein synthesis and degradation, and in addition to synthesis, proteolysis is also an important pathway. In fish, ubiquitin-mediated proteolysis is one of the important protein degradation pathways in cells, and this mechanism mainly involves the binding of small ubiquitin proteins to proteins to form ubiquitin–protein complexes, which are then recognized and degraded by degradative enzymes [54]. In ubiquitin-mediated protein degradation, the binding of ubiquitin to target proteins requires the involvement of a series of enzymes such as ubiquitin ligases (E1), ubiquitin carrier proteins (E2), and ubiquitin-conjugated target proteins (E3). In this study, the ubiquitin-mediated proteolysis signaling pathway was significantly enriched, and the expression of 22 of 27 genes, including key components of E1, E2, and E3 ubiquitin ligases, was significantly downregulated (Table S3).

The mTOR signaling pathway is a major regulatory molecular signaling pathway for cell growth and metabolism, which promotes anabolic processes, such as ribosome biogenesis and the synthesis of proteins, amino acids, etc., and inhibits catabolic processes [55]. mTORC1 plays a central role in the regulation of these processes and promotes protein synthesis, mainly by phosphorylating S6K1 and 4EBP. mTORC1 directly phosphorylates S6K1 and activates several substrates (e.g., eIF4B) that enhance the translation efficiency of mRNA, thereby promoting protein synthesis [56]. At the same time, TSC1/2, the most important protein involved in regulating mTORC1 activity, is also essential. In this experiment, the mTOR signaling pathway was significantly enriched, with genes such as S6K1, 4EBP, eIF4B, TSC1, and TSC2 significantly upregulated in the wild group (Table S4). In addition, collagen, as a major component in connective tissue, is likewise regulated by the mTOR signaling pathway [57]. In this experiment, multiple types of collagen, such as I, II, IV, and VI, in the ECM–receptor interaction pathway were significantly upregulated in wild *M. albus* (Table S6). This is consistent with the results of Yang et al. [28], who concluded that there is a strong correlation between mTOR and muscle texture quality.

The peroxisome proliferators-activated receptor (PPAR) pathway regulates adipocyte differentiation, lipid metabolism, glucose metabolism, insulin sensitivity, and other physiological processes [58]. RXRα, ACSL, CPT-1, FABP, and FATP, which are key genes of the lipid metabolism, were significantly upregulated in the PPAR signaling pathway (Table S6). The high expression of the above genes could increase fatty acid oxidation, enhance triglyceride lipolysis, inhibit the deposition of lipids in the organism, and improve the content of n-3PUFAs, which suggests that the PPAR signaling pathway plays a key role in the lipid metabolism of the wild *M. albus*.

Based on the above transcriptome results, it is hypothesized that the differences in muscle mechanisms between wild and farmed *M. albus* are partially attributed to the mTOR and PPAR signaling pathways. mTOR, on the one hand, regulates ribosomes and other protein-synthesis-related pathways to increase protein synthesis and promote muscle growth and development. On the other hand, it regulates ubiquitin-mediated proteolysis and proteolysis-related gene pathways. Studies have reported that the stimulation of nutrients, energy, and stress signals can activate the mTOR signaling pathway [59], which again demonstrated that dietary composition and the environment are the main reasons for the flesh quality differences between farmed and wild fish. Furthermore, the PPAR signaling pathway may affect the main pathway of the lipid metabolism by improving fatty acid oxidation and increasing TG lipolysis. In addition to these pathways, immune-related pathways may have a greater influence on wild and farmed muscle quality. The NOD-like receptor signaling pathway, antigen processing and presentation, and the RIG-I-like receptor signaling pathway are also significantly enriched with immune-related pathways in transcriptomics. However, there are fewer studies focused on the link between immune-related pathways and flesh quality in fish, so further research is needed.

## 5. Conclusions

Overall, the present research showed significant differences in flesh quality between wild and farmed *M. albus*, and the main manifestations were that wild *M. albus* had a higher flesh nutritive composition, reflected in crude protein, crude lipid, and amino acid and fatty acid compositions, and muscle texture, including hardness, myofiber density, and water holding capacity, than cultured *M. albus*, attributed partly to muscle development, the extracellular matrix, lipid metabolism, amino acid metabolism, and immune-related pathways, such as mTOR, PPAR, NOD-like receptor, and RIG-I-like receptor signaling pathways. These would provide theoretical references for the nutrient regulation of the flesh quality of farmed *M. albus*.

## Figures and Tables

**Table 1:** Muscle proximate composition (g/kg, wet weight) of the wild and farmed *M. albus*.

Index	Farmed	Wild	*p*-Value
Moisture	709.1 ± 6.1 ^a^	684.5 ± 7.6 ^b^	0.030 *
Crude protein	253.6 ± 5.5 ^b^	283.1 ± 4.8 ^a^	0.002 **
Crude lipid	14.7 ± 0.5 ^a^	13.1 ± 0.2 ^b^	0.013 *
Crude ash	20.1 ± 0.3 ^a^	16.3 ± 0.2 ^b^	0.010 *
Collagen	9.39 ± 0.11 ^b^	13.12 ± 0.15 ^a^	0.005 **

Values marked with asterisks are significantly different (* *p* < 0.05 and ** *p* < 0.01). Values in the same column with different superscript alphabets indicate significant differences.

**Table 2:** Muscle amino acid composition (g/kg, dry matter) of the wild and farmed *M. albus*.

Index	Farmed	Wild	*p*-Value
Arginine	44.45 ± 0.4	47.05 ± 1.7	0.136
Histidine	42.58 ± 0.4 ^b^	48.33 ± 1.6 ^a^	0.019 *
Isoleucine	18.42 ± 1.1	20.41 ± 1.4	0.193
Leucine	11.15 ± 1.5	13.04 ± 1.3	0.219
Lysine	43.91 ± 0.3 ^b^	50.44 ± 1.4 ^a^	0.008 **
Threonine	124.36 ± 2.5	124.67 ± 5.3	0.967
Valine	0.91 ± 0.0	1.04 ± 0.1	0.055
Methionine	15.81 ± 0.1 ^b^	17.78 ± 0.7 ^a^	0.031 *
Phenylalanine	17.62 ± 0.1	18.94 ± 1.1	0.210
EAAs	319.21 ± 3.9	341.71 ± 10.9	0.168
Aspartate	66.25 ± 0.5 ^b^	75.62 ± 1.8 ^a^	0.006 **
Serine	48.75 ± 2.6 ^a^	31.89 ± 1.4 ^b^	0.002 **
Glycine	5.21 ± 0.1 ^b^	5.86 ± 0.1 ^a^	0.004 **
Alanine	18.32 ± 0.1 ^b^	20.61 ± 0.8 ^a^	0.036 *
Tyrosine	103.15 ± 0.2 ^b^	114.64 ± 4.4 ^a^	0.039 *
Cysteine	21.23 ± 1.1 ^b^	25.70 ± 1.7 ^a^	0.041 *
Glutamate	112.82 ± 0.4 ^b^	131.49 ± 3.5 ^a^	0.006 **
Proline	59.22 ± 2.9 ^b^	67.58 ± 3.2 ^a^	0.035 *
NEAAs	434.02 ± 9.0	473.39 ± 10.8	0.100
TAAs	754.17 ± 5.0 ^b^	815.10 ± 16.9 ^a^	0.045 *

Values marked with asterisks are significantly different (* *p* < 0.05, and ** *p* < 0.01). Abbreviations: EAAs, essential amino acids; NEAAs, non-essential amino acids; TFAAs, total amino acids. Values in the same column with different superscript alphabets indicate significant differences.

**Table 3:** Fatty acids profile (percentage of fatty acids, %) of the wild and farmed *M. albus*.

Index	Farmed	Wild	*p*-Value
Undecanoic acid (C11:0)	—	0.64 ± 0.04	0.000 **
Lauric acid (C12:0)	1.21 ± 0.02 ^b^	1.52 ± 0.12 ^a^	0.044 *
Tridecanoic acid (C13:0)	—	0.32 ± 0.03	0.001 **
Myristic acid (C14:0)	3.51 ± 0.02 ^a^	2.61 ± 0.20 ^b^	0.010 *
Pentadecanoic acid (C15:0)	—	1.22 ± 0.06	0.000 **
Palmitic acid (C16:0)	23.42 ± 1.94 ^b^	33.66 ± 0.99 ^a^	0.003 **
Stearic acid (C18:0)	11.85 ± 1.23 ^b^	14.89 ± 0.06 ^a^	0.018 *
Tricosanoic acid (C23:0)	4.85 ± 0.25	5.52 ± 0.34	0.105
Total saturated fatty acids (SFAs)	44.84 ± 0.99 ^b^	60.39 ± 0.39 ^a^	0.000 **
Palmitoleic acid (C16:1)	8.51 ± 0.27	7.48 ± 0.47	0.071
Cis-10-Heptadecenoic acid (C17:1)	1.18 ± 0.02	—	0.000 **
Oleic acid (C18:1)	14.45 ± 1.14 ^a^	7.08 ± 0.09 ^b^	0.001 **
Nervonic acid (C24:1)	1.12 ± 0.2 ^b^	1.59 ± 0.04 ^a^	0.024 *
Total monounsaturated fatty acids (MUFAs)	25.25 ± 0.61 ^a^	16.16 ± 0.61 ^b^	0.001 **
Linoleic acid (C18:2)	21.9 ± 0.66 ^a^	8.8 ± 0.2 ^b^	0.000 **
Arachidonic acid (C20:4)	3.32 ± 0.69 ^b^	6.81 ± 0.01 ^a^	0.000 **
n-6 polyunsaturated fatty acids (n-6 PUFAs)	25.22 ± 0.4	15.61 ± 0.21	0.000 **
Linolenic acid (C18:3)	2.28 ± 0.01 ^a^	1.62 ± 0.04 ^b^	0.000 **
Eicosapentaenoic acid (C20:5)	1.20 ± 0.04 ^b^	2.21 ± 0.11 ^a^	0.001 **
Docosahexaenoic acid (C22:6)	—	1.80 ± 0.03	0.000 **
n-3 polyunsaturated fatty acids (n-3 PUFAs)	3.48 ± 0.01 ^b^	5.63 ± 0.08 ^a^	0.000 **
Total polyunsaturated fatty acids (PUFAs)	28.7 ± 0.39 ^a^	21.24 ± 0.22 ^b^	0.000 **
n-3/n-6	0.14 ± 0.00 ^b^	0.36 ± 0.01 ^a^	0.000 **

Values marked with asterisks are significantly different (* *p* < 0.05, and ** *p* < 0.01). “—” represents that fatty acid was not detected. Values in the same column with different superscript alphabets indicate significant differences

**Table 4:** Water holding capacity, texture profile, and muscle morphology of the wild and farmed *M. albus*.

Index	Farmed	Wild	*p*-Value
Water holding capacity			
Thawing loss (%)	15.44 ± 1.27 ^a^	9.03 ± 1.79 ^b^	0.007 **
Drip loss (%)	20.84 ± 2.15 ^a^	14.84 ± 3.02 ^b^	0.047 *
Steaming loss (%)	18.05 ± 1.77 ^a^	14.97 ± 0.92 ^b^	0.029 **
Boiling loss (%)	14.44 ± 1.56 ^a^	7.84 ± 2.08 ^b^	0.006 *
Texture profile			
Hardness (gf)	236.20 ± 31.46 ^b^	339.65 ± 36.28 ^a^	0.010 *
Springiness	0.68 ± 0.02 ^b^	0.93 ± 0.03 ^a^	0.001 **
Cohesiveness	0.42 ± 0.04 ^b^	0.53 ± 0.04 ^a^	0.001 **
Gumminess (gf)	89.63 ± 11.88 ^b^	146.69 ± 13.61 ^a^	0.001 **
Chewiness (gf)	67.84 ± 12.94 ^b^	106.01 ± 11.55 ^a^	0.010 *
Resilience	0.18 ± 0.02 ^b^	0.24 ± 0.02 ^a^	0.001 **
Muscle morphology			
Muscle fiber diameter (μm)	85.41 ± 5.10 ^a^	59.10 ± 3.11 ^b^	<0.001 **
Muscle fiber density (number of fibers/mm^2^)	246.14 ± 18.33 ^b^	358.02 ± 22.22 ^a^	<0.001 **

Values marked with asterisks are significantly different (* *p* < 0.05, and ** *p* < 0.01). Values in the same column with different superscript alphabets indicate significant differences

**Table 5:** Detailed information of significantly enriched KEGGs signaling pathway in the wild and farmed groups.

Pathway ID	Description	Number	*p*-Value
map04141	Protein processing in endoplasmic reticulum	61	1.30 × 10^−13^
map03010	Ribosome	42	5.86 × 10^−12^
map05171	Coronavirus disease—COVID-19	65	2.06 × 10^−8^
map05415	Diabetic cardiomyopathy	46	1.04 × 10^−4^
map05012	Parkinson’s disease	53	7.33 × 10^−4^
map03250	Viral life cycle—HIV-1	18	8.38 × 10^−4^
map03013	Nucleocytoplasmic transport	24	1.07 × 10^−3^
map00260	Glycine, serine, and threonine metabolism	13	1.66 × 10^−3^
map00513	Various types of N-glycan biosynthesis	13	1.99 × 10^−3^
map04621	NOD-like receptor signaling pathway	34	2.16 × 10^−3^
map04217	Necroptosis	33	2.71 × 10^−3^
map04216	Ferroptosis	15	3.20 × 10^−3^
map05014	Amyotrophic lateral sclerosis	64	3.56 × 10^−3^
map03320	PPAR signaling pathway	18	4.25 × 10^−3^
map05020	Prion disease	53	4.41 × 10^−3^
map05022	Pathways of neurodegeneration—multiple diseases	84	4.84 × 10^−3^
map04512	ECM–receptor interaction	22	5.13 × 10^−3^
map04612	Antigen processing and presentation	18	6.01 × 10^−3^
map03060	Protein export	7	7.16 × 10^−3^
map04714	Thermogenesis	43	7.21 × 10^−3^
map00510	N-Glycan biosynthesis	13	9.31 × 10^−3^
map04974	Protein digestion and absorption	28	9.73 × 10^−3^
map05134	Legionellosis	18	1.37 × 10^−2^
map04120	Ubiquitin-mediated proteolysis	27	1.50 × 10^−2^
map00561	Glycerolipid metabolism	16	1.52 × 10^−2^
map05010	Alzheimer’s disease	68	1.62 × 10^−2^
map04614	Renin–angiotensin system	7	2.08 × 10^−2^
map04114	Oocyte meiosis	24	2.18 × 10^−2^
map04150	mTOR signaling pathway	31	2.49 × 10^−2^
map04711	Circadian rhythm—fly	5	2.74 × 10^−2^
map04910	Insulin signaling pathway	29	3.10 × 10^−2^
map05417	Lipid and atherosclerosis	40	3.36 × 10^−2^
map05162	Measles	27	3.44 × 10^−2^
map04137	Mitophagy—animal	16	3.46 × 10^−2^
map04212	Longevity regulating pathway—worm	16	3.46 × 10^−2^
map04622	RIG-I-like receptor signaling pathway	14	4.01 × 10^−2^
map03015	mRNA surveillance pathway	17	4.37 × 10^−2^
map04146	Peroxisome	17	4.37 × 10^−2^
map05131	Shigellosis	46	4.72 × 10^−2^

## Data Availability

The original contributions presented in the study are included in the article and Supplementary Materials, further inquiries can be directed to the corresponding author.

## Supplementary Materials

The following supporting information can be downloaded at: https://www.mdpi.com/article/10.3390/foods13111751/s1, Table S1: Primers used for quantitative real-time PCR verification; Table S2: The differentially expressed genes involved in ribosome; Table S3: The differentially expressed genes involved in ubiquitin-mediated proteolysis; Table S4: The differentially expressed genes involved in mTOR signaling pathway; Table S5: The differentially expressed genes involved in ECM–receptor interaction; Table S6: The differentially expressed genes involved in PPAR signaling pathway.
