# Supplementary material for: Biochemical, Histological, and Transcriptomic Analyses Reveal Underlying Differences in Flesh Quality between Wild and Farmed Ricefield Eel (Monopterus albus)
Source: Foods. 2024 Jun 3;13(11):1751. doi: 10.3390/foods13111751 (PMC11171622; doi:10.3390/foods13111751)
Supplement: Supplementary file 1 [file foods-13-01751-s001.zip › foods-2994852-supplementary.pdf]

Table S1 Primers used for quantitative real-time PCR verification

Gene	Primer sequence	Accession number
rpl-17	F-AGAAATGCCCCATCTCCA<br>R-CCCTGTCTCCGTCTTGTTG	XM_020587712.1
col1a1	F:CTTTAAGTGTACCATGCGCTGT<br>R:ATTTGCCGGTTTTCACGGGT	XM_020625331.1
s6k1	F:TCAGGATACCACTATGGAGTTCAC<br>R:TACAGCGGCGTTCACACTTG	XM_020612395.1
fabp2	F:GGCATTGACCGCCATCTCTT<br>R:TGTCTCGCCAGGTCAAACAG	XM_020602138.1
acsl4	F:ATTGTGGTGGACTGGTTG<br>R:CTACATGAGCACCAAGACTG	XM_020591041.1
ube3a	F:ACGCACACAAACAATCTCAAGT<br>R:GCATGGGGCAAGACAGTCA	XM_020588829.1
ube2b	F:GTTATCAGGTGGCGATGGAG<br>R:GTAGACTCGTATGGGTTGTAAGG	XM_020611979.1

rpl-17, ribosomal protein L17; *col1a1*, collagen alpha-1(I); *fabp2*, fatty acid binding protein 2; *s6k1*, ribosomal protein S6 kinase beta-1; *acsl4*, acyl-CoA synthetase long-chain family member 4; *ube3a,* ubiquitin-protein ligase E3A; *ube2b*, ubiquitin conjugating enzyme E2 B.

Table S2 The differentially expressed genes involved in Ribosome

Gene name	Gene description	Regulate	FC	P-value
rpl10a	ribosomal protein L10a	up	2.52	1.40E-02
rps27a	ribosomal protein S27a	up	2.04	4.10E-02
LOC109956091	60S ribosomal protein L36a	up	2.07	2.53E-02
rpl6	ribosomal protein L6	up	2.21	2.90E-02
rps13	ribosomal protein S13	up	2.42	1.89E-02
mrpl33	mitochondrial ribosomal protein L33	up	2.03	4.46E-02
rplp2	ribosomal protein lateral stalk subunit P2	up	3.06	1.86E-03
rps2	ribosomal protein S2, transcript variant X2	up	2.39	1.05E-02
rpl38	ribosomal protein L38	up	2.48	1.94E-02
LOC109963109	60S ribosomal protein L19-like	up	2.52	6.89E-03
rpl31	ribosomal protein L31, transcript variant X2	up	2.22	1.81E-02

rpl17	ribosomal protein L17	up	2.06	4.55E-02
rps6	ribosomal protein S6	up	2.16	3.71E-02
rpl14	ribosomal protein L14	up	2.23	9.86E-03
LOC109967421	40S ribosomal protein S27-like	up	13.73	1.13E-02
rpl29	ribosomal protein L29	up	2.34	1.28E-02
rps25	ribosomal protein S25	up	2.11	3.84E-02
rps7	ribosomal protein S7, transcript variant X1	up	2.64	8.80E-03
rps3a	ribosomal protein S3A	up	2.48	1.21E-02
rpl23	ribosomal protein L23	up	2.09	2.69E-02
rpl27	ribosomal protein L27	up	2.40	1.32E-02
rpl13a	ribosomal protein L13a	up	3.09	8.11E-04
rps8	ribosomal protein S8	up	2.57	1.01E-02
mrpl21	mitochondrial ribosomal protein L21	down	0.41	3.40E-03
rpl24	ribosomal protein L24	up	2.21	1.93E-02
rps19	ribosomal protein S19	up	2.61	7.94E-03
uba52	ubiquitin A-52 residue ribosomal protein fusion product 1	up	2.15	1.42E-02
rps11	ribosomal protein S11, transcript variant X1	up	2.46	6.43E-03
rpl12	ribosomal protein L12	up	2.66	1.13E-03
rps12	ribosomal protein S12	up	2.64	1.31E-03
fau	FAU, ubiquitin like and ribosomal protein S30 fusion	up	2.14	1.88E-02
rpl37	ribosomal protein L37	up	2.27	1.07E-02
rpl18	ribosomal protein L18	up	2.09	4.99E-02
rps20	ribosomal protein S20	up	2.78	2.20E-04
rps26	ribosomal protein S26	up	2.11	3.09E-02
rpl23a	ribosomal protein L23a	up	2.14	1.44E-02
rps23	ribosomal protein S23	up	2.91	4.78E-04
LOC109969138	40S ribosomal protein S17, transcript variant X1	up	2.05	3.61E-02
rps24	ribosomal protein S24, transcript variant X1	up	2.16	3.83E-02
rpl27a	ribosomal protein L27a	up	2.12	2.70E-02
mrpl16	mitochondrial ribosomal protein L16, transcript variant X1	down	0.46	1.05E-02
rpsa	ribosomal protein SA	up	2.17	3.78E-02

Table S3 The differentially expressed genes involved in Ubiquitin mediated proteolysis

Gene name	Gene description	Regulate	FC	P-value
trim37	tripartite motif containing 37, transcript variant X1	up	2.67	3.52E-02
rps27a	ribosomal protein S27a	up	2.04	4.10E-02
ube2i	ubiquitin conjugating enzyme E2 I, transcript variant X1	down	0.22	4.19E-02
LOC109953363	ubiquitin-protein ligase E3A	down	0.49	8.51E-03
anapc11	anaphase promoting complex subunit 11	down	0.27	2.58E-04

LOC109958966	ubiquitin-like modifier-activating enzyme 1	down	0.24	1.19E-02
syvn1	synoviolin 1, transcript variant X1	down	0.30	8.46E-03
fbxw11	F-box and WD repeat domain containing 11, transcript variant X1	down	0.36	4.87E-03
cul1	cullin 1, transcript variant X4	down	0.31	5.29E-04
ddb1	damage specific DNA binding protein 1	up	2.21	4.90E-02
herc2	HECT and RLD domain containing E3 ubiquitin protein ligase 2	down	0.40	4.45E-02
uba1	ubiquitin like modifier activating enzyme 1, transcript variant X1	down	0.47	2.11E-02
ube2b	ubiquitin conjugating enzyme E2 B, transcript variant X1	down	0.45	5.13E-03
LOC109973961	ubiquitin-conjugating enzyme E2 Q2-like, transcript variant X1	down	0.35	2.13E-03
LOC109962584	probable E3 ubiquitin-protein ligase HERC4, transcript variant X2	down	0.20	3.07E-03
trip12	thyroid hormone receptor interactor 12, transcript variant X5	down	0.34	3.31E-03
LOC109963682	ubiquitin-conjugating enzyme E2 E2-like, transcript variant X1	down	0.27	3.14E-02
LOC109963674	ubiquitin-conjugating enzyme E2 E1, transcript variant X1	down	0.41	2.76E-04
uba52	ubiquitin A-52 residue ribosomal protein fusion product 1	up	2.15	1.42E-02
huwe1	HECT, UBA and WWE domain containing 1, E3 ubiquitin protein ligase	down	0.46	7.69E-03
LOC109957680	cullin-3-like	down	0.34	7.15E-04
LOC109962585	probable E3 ubiquitin-protein ligase HERC3	down	0.06	1.65E-02
LOC109970578	cullin-3-like	down	0.43	2.13E-03
cul4b	cullin 4B	down	0.26	3.13E-04
LOC109956179	F-box only protein 2-like	up	2.99	9.80E-03
cdc27	cell division cycle 27	down	0.31	3.80E-05
LOC109969285	ubiquitin-conjugating enzyme E2 R1-like	down	0.40	1.25E-02

Table S4 The differentially expressed genes involved in mTOR signaling pathway

Gene name	Gene description	Regulate	FC	P-value
LOC109954584	phosphatidate phosphatase LPIN1-like, transcript variant X1	down	0.04	7.99E-14
LOC109959759	ras-related GTP-binding protein D-like, transcript variant X1	down	0.37	7.91E-03
tsc2	tuberous sclerosis 2, transcript variant X2	up	2.49	1.30E-02
LOC109955468	GTP-binding protein Rheb	down	0.37	1.02E-02
rps6ka3	ribosomal protein S6 kinase A3, transcript variant X3	up	2.20	3.56E-03

LOC109966856	phosphatidylinositol 3-kinase regulatory subunit gamma-like, transcript variant X4	up	4.34	2.63E-02
LOC109959592	ras-related GTP-binding protein A, transcript variant X1	up	2.03	3.81E-02
fnip1	folliculin interacting protein 1, transcript variant X1	up	4.28	7.75E-04
lpin2	lipin 2, transcript variant X1	up	3.35	2.44E-03
lamtor5	late endosomal/lysosomal adaptor, MAPK and MTOR activator 5	up	7.06	3.70E-03
rps6ka6	ribosomal protein S6 kinase A6	up	2.50	2.58E-02
dvl2	dishevelled segment polarity protein 2	up	2.37	1.88E-02
clip1	CAP-Gly domain containing linker protein 1, transcript variant X1	down	0.38	1.32E-02
rps6	ribosomal protein S6	up	2.16	3.71E-02
wdr59	WD repeat domain 59	down	0.30	2.56E-04
ddit4	DNA damage inducible transcript 4	down	0.26	1.62E-02
LOC109957432	hamartin-like	down	0.41	2.43E-03
LOC109972722	segment polarity protein dishevelled homolog DVL-3-like, transcript variant X1	up	2.25	1.25E-03
gsk3b	glycogen synthase kinase 3 beta, transcript variant X1	down	0.17	2.78E-04
LOC109956162	phosphatidate phosphatase LPIN2-like, transcript variant X1	down	0.38	1.61E-02
raf1	Raf-1 proto-oncogene, serine/threonine kinase, transcript variant X2	down	0.24	1.39E-03
LOC109959930	V-type proton ATPase subunit E 1-like, transcript variant X1	up	2.39	3.18E-02
LOC109963978	sestrin-2-like, transcript variant X1	up	2.43	4.27E-02
LOC109955981	keratin, type I cytoskeletal 18-like, transcript variant X1	up	2.09	4.90E-02
akt1s1	AKT1 substrate 1	up	2.35	7.02E-04
atp6v1d	ATPase H+ transporting V1 subunit D	down	0.33	3.21E-04
LOC109966482	insulin receptor substrate 1-B-like	down	0.20	1.50E-05
pten	phosphatase and tensin homolog, transcript variant X1	down	0.47	1.52E-02
LOC109959155	GATS-like protein 3	up	2.78	1.77E-02
LOC109960282	ribosomal protein S6 kinase beta-1-like	up	2.34	3.33E-03
lamtor4	late endosomal/lysosomal adaptor, MAPK and MTOR activator 4	up	2.28	9.56E-03

Table S5 The differentially expressed genes involved in ECM-receptor interaction

Gene name	Gene description	Regulate	FC	p-value
col6a2	collagen type VI alpha 2 chain	up	2.66	1.25E-02
col4a6	collagen type IV alpha 6 chain, transcript variant X1	up	2.34	2.39E-02
LOC109971201	collagen alpha-1(II) chain-like	up	2.44	3.97E-02
LOC109971731	collagen alpha-3(VI) chain-like	up	2.11	2.85E-02

LOC109966919	syndecan-4-like	down	0.29	6.31E-03
LOC109969770	tenascin-like	up	13.08	3.82E-02
LOC109969127	integrin alpha-11-like	down	0.43	3.12E-02
col6a1	collagen type VI alpha 1 chain	up	2.91	5.90E-03
LOC109964120	fibronectin-like, transcript variant X1	down	0.43	4.12E-02
itga7	integrin subunit alpha 7, transcript variant X1	down	0.30	2.39E-05
LOC109969769	tenascin-like, transcript variant X4	down	0.18	6.96E-04
col4a5	collagen type IV alpha 5 chain, transcript variant X1	up	2.85	2.37E-02
LOC109970053	kunitz-type serine protease inhibitor BmKTT-2-like	up	3.28	3.08E-03
LOC109972354	thrombospondin-4-B-like	down	0.40	2.87E-02
LOC109957036	thrombospondin-4-B-like	up	2.35	3.77E-02
thbs3	thrombospondin 3	down	0.34	2.69E-02
LOC109974984	laminin subunit alpha-1-like	down	0.13	5.94E-05
LOC109966907	dystroglycan-like	up	2.60	4.89E-04
col1a2	collagen type I alpha 2 chain	up	2.50	2.42E-02
LOC109970071	collagen alpha-6(VI) chain-like	up	2.91	6.31E-03
itga6	integrin subunit alpha 6, transcript variant X1	up	3.09	3.11E-02
LOC109974887	collagen alpha-1(I) chain-like, transcript variant X1	up	2.84	1.57E-02

Table S6 The differentially expressed genes involved in PPAR signaling pathway

Gene name	Gene description	Regulate	FC	P-value
LOC109963008	acyl-CoA desaturase-like	up	5.35	1.36E-03
LOC109963312	very long-chain acyl-CoA synthetase-like	up	6.64	1.16E-05
LOC109955654	cytochrome P450 27C1-like, transcript variant X2	down	0.30	2.58E-03
acadm	acyl-CoA dehydrogenase, C-4 to C-12 straight chain	down	0.47	2.38E-03
LOC109956993	long-chain-fatty-acid--CoA ligase 1-like	up	2.65	3.68E-02
LOC109963311	very long-chain acyl-CoA synthetase-like	up	15.35	8.41E-11
acsl4	acyl-CoA synthetase long-chain family member 4, transcript variant X3	up	3.30	1.28E-02
LOC109968035	fatty acid-binding protein, brain-like	up	39.23	3.88E-06
LOC109967077	carnitine O-palmitoyltransferase 1, liver isoform-like	up	2.46	1.82E-02
LOC109963313	very long-chain acyl-CoA synthetase-like	up	30.29	4.15E-09
LOC109974250	perilipin-2-like, transcript variant X1	down	0.45	3.47E-02
angptl4	angiopoietin like 4	down	0.22	1.50E-04
LOC109966867	angiopoietin-related protein 4-like	down	0.17	1.22E-02
LOC109959447	sorbin and SH3 domain-containing protein 1-like, transcript variant X1	up	2.29	5.49E-03
LOC109971684	retinoic acid receptor RXR-beta-A-like	up	2.28	3.45E-04
LOC109960743	hydroxymethylglutaryl-CoA synthase, cytoplasmic-like, transcript variant X1	down	0.27	4.78E-03
acaa1	acetyl-CoA acyltransferase 1	up	2.25	4.75E-05
fabp2	fatty acid binding protein 2	up	2.87	4.68E-04
